# Spatio-temporal dynamics of neocortical presynaptic terminal development using multi-photon imaging of the corpus callosum *in vivo*

**DOI:** 10.1038/s41598-019-50431-6

**Published:** 2019-10-01

**Authors:** Teresa A. Evans, Luke A. Bury, Alex Y. Huang, Shasta L. Sabo

**Affiliations:** 1 Departments of Pharmacology and Neuroscience, Case Western Reserve University School of Medicine, Cleveland, USA; 2 Department of Pediatrics, Case Western Reserve University School of Medicine, Cleveland, USA; 3 Department of Biology, Central Michigan University, Mount Pleasant, USA; 4 Present Address: Department of Pediatrics, Stanford University, Stanford, USA

**Keywords:** Synaptic development, Neural circuits

## Abstract

Within the developing central nervous system, the dynamics of synapse formation and elimination are insufficiently understood. It is ideal to study these processes *in vivo*, where neurons form synapses within appropriate behavioral and anatomical contexts. *In vivo* analysis is particularly important for long-range connections, since their development cannot be adequately studied *in vitro*. The corpus callosum (CC) represents a clinically-relevant long-range connection since several neurodevelopmental diseases involve CC defects. Here, we present a novel strategy for *in vivo* longitudinal and rapid time-lapse imaging of CC presynaptic terminal development. In postnatal mice, the time-course of CC presynaptic terminal formation and elimination was highly variable between axons or groups of axons. Young presynaptic terminals were remarkably dynamic – moving, dividing to generate more boutons, and merging to consolidate small terminals into large boutons. As synaptic networks matured, presynaptic mobility decreased. These rapid dynamics may be important for establishing initial synaptic contacts with postsynaptic partners, refining connectivity patterns or modifying synapse strength during development. Ultimately, this *in vivo* imaging approach will facilitate investigation of synapse development in other long-range connections and neurodevelopmental disease models.

## Introduction

The corpus callosum (CC) comprises a bundle of long-range projections that are responsible for reciprocal interactions between cerebral hemispheres^1^. Inter-hemispheric communication is necessary for integration of the hemispheres and for lateralized functions, such as language and facial recognition. Abnormal callosal connectivity is linked to neurodevelopmental diseases, such as schizophrenia, autism, childhood PTSD, ADHD, dyslexia, epilepsy and Tourette syndrome^2,3^.

Callosally projecting cortical neurons reside in cortical layers 2/3 and 5. Layer 2/3 pyramidal neurons comprise ~80% of callosal neurons, with the majority deriving from layer 3^4^. After crossing the midline, callosal axons project most strongly to layers 1–3 and 5 within the contralateral hemisphere^4–6^. Within layer 1, some axons turn and extend radially for several millimeters^7^.

Callosal axons form excitatory synapses with spines of pyramidal neurons^6,8–11^. These synapses are most abundant in supragranular layers^10–12^. Interestingly, a large majority of pyramidal neurons that reside in layers 2/3 and 5 receive callosal input, indicating that the prevalence and influence of callosal synapses is substantial^13^. Examination of swellings within filled cells suggests that boutons appear either as terminal boutons at the ends of axonal projections or as *en passant* synapses along the lengths of axons^7,12^. Terminal boutons are found predominantly in Layer 1^7^, while *en passant* synapses appear throughout targeted layers^7^.

CC axons develop during prenatal and early postnatal periods^6,14–20^. CC projections are initially established exuberantly then refined to their mature patterns^15,21^. *In vivo* imaging of CC axons has revealed both elaboration of branches in supragranular layers and pruning of axon terminal branches between postnatal days 10–14 (P10-14)^6,20^. Overall, CC axonal arbors reach their mature region- and layer-specific patterns toward the end of the second postnatal week in mice and rats^18,19,22,23^.

While CC axon growth and guidance have been extensively studied^1^, the mechanisms and dynamics of callosal synapse development remain underexplored. By the time axons have grown into contralateral cortex, synapses have already begun to form^24^. Boutons appear first in infragranular layers followed by supragranular layers^25^. During postnatal development, the number of synapses initially increases with age^4^. This period of synapse growth is then followed by a decline in CC synapse number to adult levels^4,7,24^. By the third postnatal week, presynaptic terminals mature, shifting from a low to high probability of release^26^.

Understanding the dynamics of synapse formation is important since synaptogenesis is a critical step in the establishment of precise neuronal circuits. Dynamics of neocortical synapse development have primarily been investigated *in vitro*^27^, where neurons forming long-range connections lack their native networks or have severed axons. *In vitro* experiments also preclude investigation of how synapse development is influenced by sensory experience, behavior or communication from non-cortical brain areas. *Ex vivo* studies of synapse development have utilized fixed tissue and comparisons across animals, but variability and neural circuit complexity necessitates longitudinal data from the same neurons to understand the dynamics of synapse development within intact neural networks. *In vivo* studies have primarily focused on dendritic spines^28–31^, but many nascent synapses may be invisible with this strategy since substantial cortical synapse formation occurs with alternative postsynaptic structures, such as filopodia and dendrite shafts^32^. Furthermore, in these studies, all spines of a labeled neuron are visualized, so it is unclear which synapses are part of a particular long-range projection, such as the CC.

Here, we applied *in vivo* imaging through cranial windows to examine the development of presynaptic terminals within CC axons during the second and third weeks of postnatal development. To do so, we labeled presynaptic terminals of CC projection neurons with synaptophysin-tdTomato by using unilateral expression of Cre-GFP in a Cre-dependent synaptophysin-tdTomato mouse. To analyze the dynamics of synapse formation and elimination, presynaptic terminals were imaged repeatedly over both short (every 30 s for up to 30 minutes) and long (every 24 h for up to 14 days) timescales. During this period of postnatal development, the time-course and magnitude of changes in presynaptic terminal density were highly variable across imaged fields of view. In addition, we did not observe distinct phases of synapse formation and elimination in the population, but periods of formation and elimination were observed in individual imaged fields. Throughout development, presynaptic terminals were surprisingly dynamic – moving rapidly, dividing to form more terminals, and consolidating to form fewer terminals. Thus, we have established a powerful strategy for studying development of synapses formed by long-range projections and demonstrated that this approach can be applied to examine both short-term and long-term changes in synaptogenesis.

## Results

**Figure 1:**
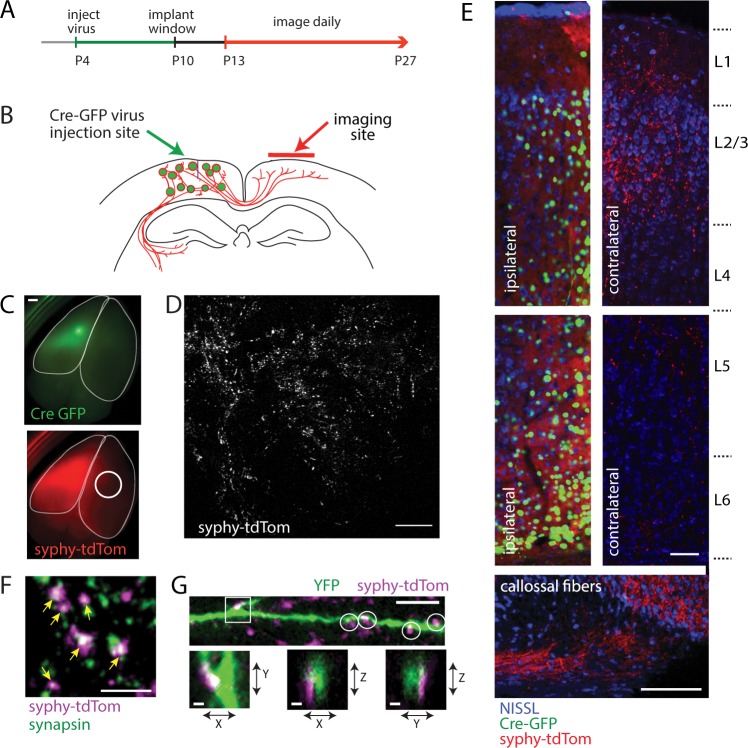
Strategy for *in vivo* live imaging of corpus callosal presynaptic development. (**A**) Experimental timeline. Virus was injected at P4, cranial windows were implanted at P10, and images were collected of the same volume of cortex from P13-27 (longitudinal imaging) or P13-21 (time-lapse imaging). (**B**) Diagram of unilateral injection of AAV1-hSyn1-Cre-GFP (*left hemisphere*, *green*) into floxed synaptophysin-tdTomato mice and contralateral imaging of presynaptic terminals formed by callosal axons (*right hemisphere*, *red*). (**C**) *Top*, Unilateral expression of Cre-GFP (*green*). *Bottom*, synaptophysin-tdTomato expression (*red*) is observed within homotypic contralateral cortex (*circle*, site of imaging*; outlines*, cortical perimeter). Scale bar, 0.5 mm. (**D**) Higher magnification view of the imaging site. Image shown is a Z-projection that represents a volume of supragranular cortex, with X and Y dimensions running parallel to the cortical surface. Scale bar, 100 µm. (**E**) Histology showing Nissl (*blue*), Cre-GFP (*green*) and synaptophysin-tdTomato (*red*) in a coronal section of the virus-injected hemisphere (*left*), CC axons entering contralateral cortex (*bottom*), and synaptophysin-tdTomato-labeled boutons in contralateral cortex (*right*). Upper and lower cortical layers are presented as separate panels since they were collected in separate images from the same section of cortex (using the same imaging parameters). Scale bars, 50 µm and 100 µm. (**F**) Synaptophysin-tdTomato (*magenta*) and endogenous synapsin, identified by immunofluorescent labeling (*green*) colocalize (*arrows*). Scale bar, 10 µm. (**G**) Synaptophysin-tdTomato-labeled terminals (*magenta*) in close association (*circles*, *box*) with YFP-filled dendrites (*green*) in layer 2/3 of contralateral cortex of double-transgenic Thy1-YFP/synaptophysin-tdTomato mice, supporting localization at synapses. Scale bar, 10 µm. Magnified X-Y, X-Z and Y-Z planes are shown for the boxed area. Scale bars, 1 µm. Images in C, E and F are from mice at age P29, while images for D and G were collected at P27 and P26, respectively.

To investigate the dynamics of CC synapse development *in vivo*, we developed a strategy for longitudinal intravital imaging of presynaptic terminals in postnatal mice. To label CC presynaptic terminals, AAV1 encoding Cre-GFP was injected into one hemisphere of neonatal stop-flox synaptophysin-tdTomato mice^33^ (Fig. 1A–C). At the injection site, neurons expressed Cre-GFP and synaptophysin-tdTomato (Fig. 1B–E). In contralateral cortex, synaptophysin selectively labeled CC boutons of axons traversing the corpus callosum (Fig. 1B,C,E), with only negligible labeling outside of these puncta. We did not observe Cre-GFP expression in the thalamus contralateral to the virus injection (not shown). Synaptophysin-tdTomato specifically labeled presynaptic terminals since it co-localized with endogenous synapsin, and these terminals were likely associated with postsynaptic partners since they frequently appeared in close apposition to dendrites and dendritic spines (Fig. 1F,G and Supplementary Fig. S1). However, we refer to synaptophysin-positive puncta as presynaptic terminals (or boutons) here since presynaptic sites that lack postsynaptic partners have been reported for several types of axons^34–37^. It is worth noting that presynaptic terminals that lack postsynaptic partners are fully-functional, releasing neurotransmitter in response to depolarization, and are frequently recruited to contacts with dendrites to rapidly form synapses^34,36^.

Labeled CC terminals were imaged through cranial windows via two-photon time-lapse imaging (Fig. 1A–D). Windows were implanted at P10, over primary somatosensory cortex. As previously reported^20^, when implanted after P8, windows did not appear to affect skull growth or animal development (Supplementary Fig. S2). CC axon branching patterns are mature and mostly stable by P12, and synapse formation is thought to occur at high rates from this age onward^1,23^. Therefore, imaging was performed daily from P13-P27. During this period of postnatal development, we analyzed longitudinal changes in presynaptic terminal density and stability within supragranular layers of cortex. In some animals, bone regrew under the mounted windows, which obscures imaging; therefore, we also imaged bone regrowth each day and ended imaging if bone growth encroached on the imaging field. In some instances, imaging began after P13 for practical reasons.

**Figure 2:**
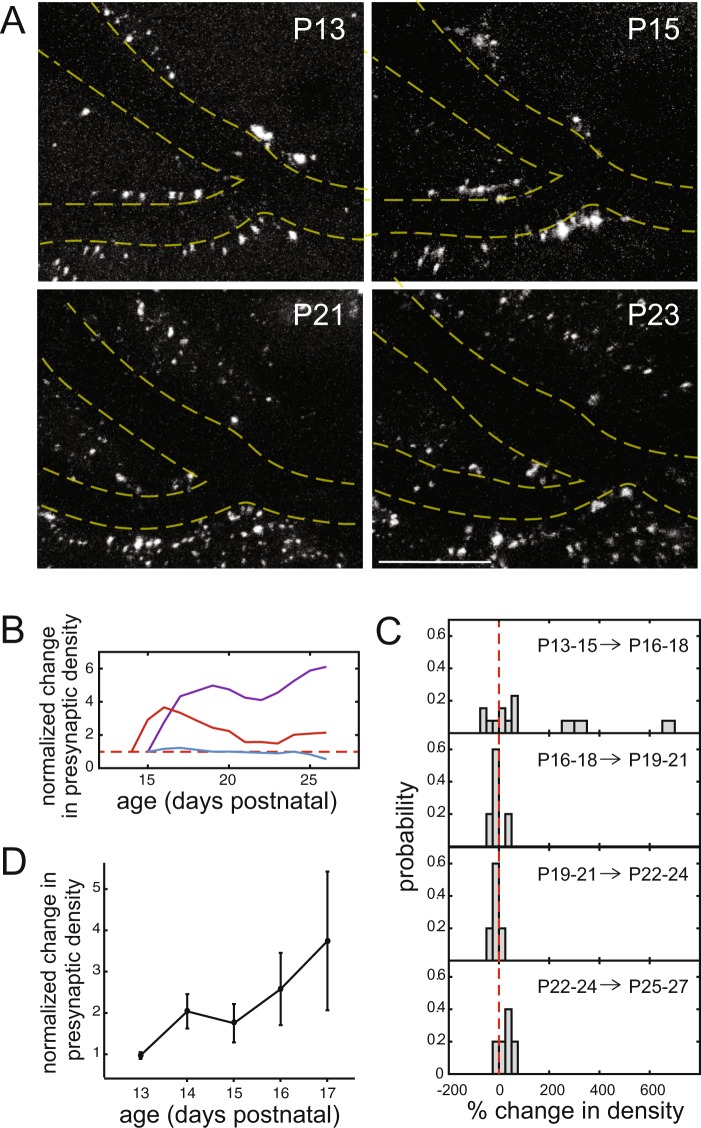
*In vivo* imaging of corpus callosal presynaptic terminals in developing neocortex: longitudinal analysis. (**A**) Low-magnification images of synaptophysin-tdTomato (*white*). Blood vessels (*dashed yellow lines*) served as fiduciary marks, allowing imaging of the same brain volume on successive days. Scale bar, 50 µm. Representative images are shown for two days at the start of imaging (P13 and P15) and the beginning of the third postnatal week (P21 and P23). The images shown are maximum intensity Z-projections of the imaged volume. (**B**) Longitudinal changes in presynaptic terminal density, normalized to the initial value on the first day of imaging. Each line represents the fold change in bouton density in one imaged region of contralateral cortex. CC synapse density dynamics were variable, even within different fields-of-view in the same brain, as illustrated by the *purple* and *red* lines, which were obtained by imaging two distinct CC fields in the same animal. In some cases, presynaptic terminal density was remarkably stable throughout this period of development (*blue*, from a different mouse than the purple and red lines). (**C**) Probability histograms of changes in bouton density as animals matured. For each imaged region, data were binned to calculate an average density over the indicated 3-day intervals, then changes in density were calculated from one bin to the next (n = 17 regions from 9 mice, with 1-2 fields imaged per mouse). For example, the top panel presents a histogram of the changes between bin 1 (P13-15) and bin 2 (P16-18) for all regions imaged. Increases in density are represented as positive changes in density, while decreases appear as negative changes in density. Wider bars correspond to wider bins, used to accurately display low probability occurrences. Across all age groups, observed age-dependent changes in density were significant (*Z* = −3.11, *p* = 0.0019, two-sided Wilcoxon signed rank test). (**D**) When the data for all regions were pooled and averaged, presynaptic density appeared to increase over time during the most dynamic time period (P13-17; normalized to P13; n = 10 imaged regions from 5 mice, with 2 regions per mouse; χ^2^(4, 38) = 5.77, p = 0.2167, Kruskal-Wallis test with Tukey-Kramer multiple comparisons correction).

We were able to use both external and natural fiduciary cues, such as marks placed on the cortical windows and blood vessel patterns, to identify the same brain volumes for imaging on successive days (Fig. 2A and Supplementary Fig. S3). In initial experiments, we filled blood vessels with dye to aid in identification of the same region at different times; however, we found that the blood vessel dye was unnecessary since the absence of fluorescence within blood vessels was sufficient to allow repeated identification of the same brain volumes on each day of imaging. In addition, there are drawbacks to use of vessel dye: injection of a dextran-based vessel dye is another intervention for the animals, and vessel dye tends to build up over time in cells around vessels. Therefore, for the imaging presented here, we chose to avoid the use of blood vessel dye.

Daily imaging of synaptic changes at the same location (Fig. 2A and Supplementary Fig. S3) revealed that the time-course of presynaptic terminal formation and elimination was much more variable than expected by examining population averages. Some imaged fields experienced substantial increases in bouton density over time (Fig. 2B, *purple*), while others displayed little change (Fig. 2B, *blue*). In addition, some regions exhibited sequential phases of bouton formation and elimination (Fig. 2B, *red*). Even regions within the same brain experienced different dynamics of bouton growth, as demonstrated in Fig. 2B, where the time courses shown in *purple* and *red* were imaged in different locations, and presumably different axons, within the same animal.

To further analyze changes in presynaptic terminal density as mice matured, we compared the densities of synaptophysin puncta at 5 periods of development, each spanning 3 days: P13-15, P16-18, P19-21, P22-24 and P25-27. For each region imaged, we (i) measured puncta density daily, (ii) averaged these measurements for each 3-day time period, and (iii) calculated the difference between the mean densities for successive 3-day periods (e.g. P13-15 vs P16-18 and P16-18 vs P19-21). Then, the data for all mice and imaged regions were plotted (Fig. 2C). In most cases, two distinct regions were imaged in the same mouse. Because these regions were separated by enough distance that they likely represented terminals formed by different axons and with distinct postsynaptic neurons, and the variability between regions imaged within the same mouse appeared to be as high as the variability from mouse to mouse (see Fig. 2B for example), all imaged fields were treated as independent for analysis. As described above, the time course of changes in density varied for different imaged axons/regions. For several regions, increases in CC presynaptic terminal density were observed early, between P13-15 and P16-18 (Fig. 2C, *top panel*, *right of the dashed zero line*). A few regions displayed especially large increases (*wide positive bins*). For other regions, there was little to no change in density (corresponding to bins at the zero line), while others lost boutons (bins to the left of the dashed line). Later in development, bouton growth plateaued, and changes in density were more modest (Fig. 2C), as indicated by concentration of the changes to bins near zero. For all age groups, the observed changes in presynaptic density were significant (*Z* = −3.11, *p* = 0.0019, two-sided Wilcoxon signed rank test). When all the data were pooled, mean bouton density appeared to increase steadily during the initial, highly-dynamic growth period (P13-P17; Fig. 2D). However, this apparent increase in density did not reach significance (p = 0.2167, χ^2^(4, 38) = 5.77, Kruskal-Wallis test), possibly due to the high variability from axon to axon (or region to region). Interestingly, in the pooled data, we did not observe distinct phases of synapse formation and elimination (Fig. 2C), even though such phases were observed in some imaging fields (Fig. 2B, *red*).

**Figure 3:**
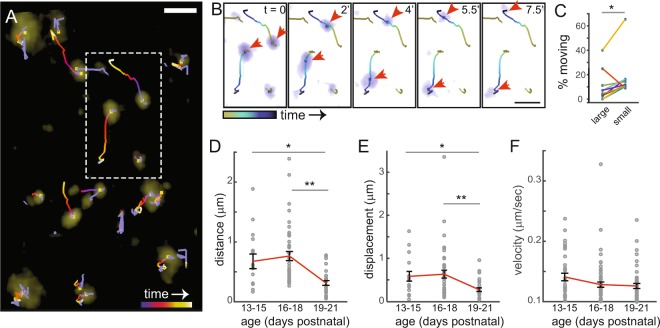
Time-lapse imaging of developing corpus callosal presynaptic terminals *in vivo*: presynaptic terminals move rapidly within the developing cortex, in an age-dependent manner. (**A**) Example of presynaptic terminal (*yellow*) movement at P14. Tracks of movement are color-coded for time (total time is 7.5 minutes). Scale bar, 5 µm. (**B**) Filmstrip of boxed area from A showing two mobile puncta (*red arrows*). Times are indicated in minutes on each panel. Scale bar, 5 µm. (**C**) Percentage of puncta that moved in the initial 5 minutes of imaging. Large puncta, apparent radius of at least 2.5 µm. Large puncta were less mobile than small puncta (repeated measures ANOVA: *F* (1, 9) = 15.39, *p = 0.0066, n = 10 movies, each from a different mouse). (**D**) Boutons traveled shorter distances with increasing age. *χ*^2^ (2, 84) = 24.17, * *p* = 0.0168; **p = 3.28 × 10^−6^. (**E**) Net displacement decreased with increasing age. *χ*^2^ (2, 84) = 14.40, *p = 0.0235; **p = 8.63 × 10^−4^; for D and E, n = 16, 42 and 29 puncta from 5, 9 and 9 movies and 3, 4 and 4 mice, respectively. (**F**) Instantaneous velocity was unchanged with age. *χ*^2^ (2, 143) = 5.52, p = 0.06; n = 35, 64, 47 movements of 16, 42 and 29 puncta. The same puncta were tracked for all measures in (**D**–**F**), and statistical comparisons were via Kruskal-Wallis tests with Dunn-Sidak multiple comparisons corrections. Data were obtained from at least 3 mice per age group.

Next, we sought to understand whether individual CC presynaptic terminals are stable during development. Imaging every 30 s for up to 30 minutes, from ages P13-21, revealed that most boutons were stable over this time course, while a population of callosal presynaptic terminals was mobile (Fig. 3A,B and Supplementary Movie 1). This observation is consistent with previous reports indicating that fully-functional presynaptic terminals can move within axons *in vitro*^34,36^. Based on these *in vitro* studies, the moving puncta might correspond to movement of an entire synapse or to nascent or “orphan” presynaptic terminals migrating to a site of contact with a dendrite^34,36^.

Upon initial inspection of time lapse movies, it appeared as though a higher percentage of small puncta were mobile when compared to larger puncta. To test this, we divided puncta into small and large groups based on their apparent diameters in the first frame of each movie. We then fit the distribution of puncta with respect to diameter by using an unbiased Gaussian mixture model with two Gaussians (Supplementary Fig. S4A), and the data were clustered based on the parameters from the fitted model (Supplementary Fig. S4B,C). From histograms of puncta sorted by the cluster analysis, we chose 2.5 μm as the threshold to sort small and large puncta from each movie then determined the percentage of small and large puncta that moved. More small puncta were mobile than large puncta (Fig. 3C**;** in 5 minutes, 17.6% +/− 5.4% and 10.0% + /− 4.0% moved, respectively; p < 0.0066), suggesting that larger puncta correspond to more stable synapses. Despite this, the percentage of terminals classified as large or small did not appear to increase with age (linear regression: y = −0.6 × +/− 20.1, R^2^ = 0.229; similar low R^2^ values were obtained with log or exponential fits to the data).

For moving puncta, a range of paths was observed, some straight and others circuitous or curved (Fig. 3A and Supplementary Movie 1). The sparseness of mobile puncta suggests that these boutons likely correspond to presynaptic terminal structures moving along axons, as described *in vitro*^34^, since changes in the underlying axon structure would be expected to displace all puncta within a moving branch. Given the small diameter of axons, nonlinear routes are expected to be a result of axon curvature or movement from one branch to another rather than axial movement within the axon.

When individual puncta were tracked over time, CC presynaptic terminals became less mobile as mice matured, traveling shorter distances with reduced net displacement at P19-21 as compared to earlier timepoints (Fig. 3D,E, Supplementary Table 1**;** only puncta that moved were included in these analyses). In contrast, instantaneous velocities were unchanged with age (Fig. 3F, Supplementary Table 1), consistent with the same number and/or type of molecular motors being active throughout development^37–39^. Interestingly, the instantaneous velocities observed here were slower than those reported for transport of synaptic vesicle precursors *in vitro*^36,40,41^. Based on the mean distance traveled and instantaneous velocity (shown in Fig. 3D,F), presynaptic puncta that moved were estimated to be mobile for less than 10% of the time (P13-15: 7.98%; P16-18: 9.93%; P19-21: 4.14%). The same puncta were tracked for each measure of mobility. Since instantaneous velocities were unchanged while distances traveled were reduced with age, one can infer that presynaptic terminals moved less often or for shorter durations within more mature axons.

**Figure 4:**
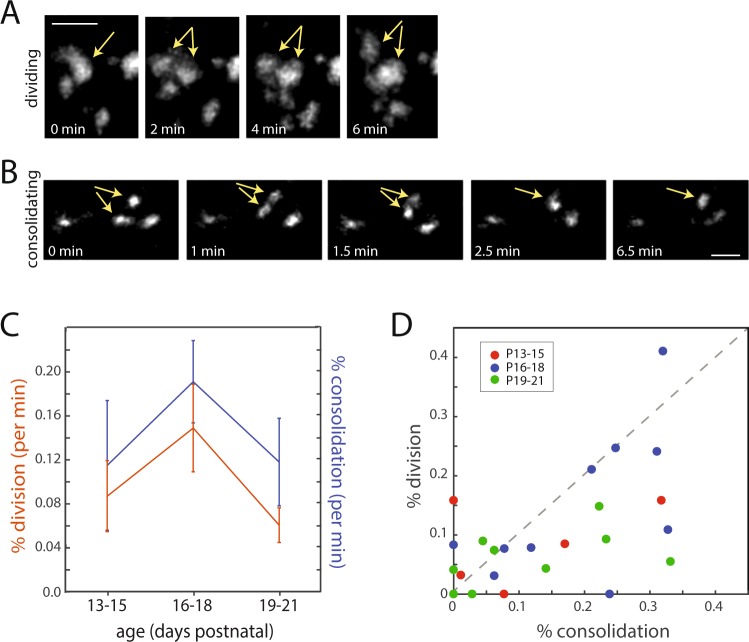
Time-lapse imaging of developing corpus callosal presynaptic terminals *in vivo*: presynaptic terminals divide and consolidate throughout synapse development. (**A**) Example of one presynaptic terminal dividing to form two separate puncta (*arrows*) at P18. Scale bar, 5 µm. (**B**) Example of two puncta consolidating to form a single punctum (*arrows*) at P18. Scale bar, 5 µm. (**C**) Quantification of dividing and consolidating puncta. Rates correspond to the percentage of puncta that either divided or consolidated in one minute of imaging. Rates of puncta division were not significantly different than rates of consolidation (*F* (1, 21) = 3.99, *p* = 0.0588, repeated measures ANOVA with age as a between factor). There were no significant age-dependent differences in division (*χ*^2^ (2, 21) = 2.65, p = 0.2657) or consolidation (*χ*^2^ (2, 21) = 2.42, p = 0.2982) via Kruskal-Wallis tests with Dunn-Sidak multiple comparisons corrections. n = 5, 10 and 9 movies at P13-15, P16-18 and P19-21, respectively. Data were obtained from at least 3 mice per age group. (**D**) Comparison of the percentage of puncta that consolidated and divided for each movie. Data points that fall above the dashed line correspond to regions (or axons) with a net increase in presynaptic terminal density from the combined effects of division and consolidation; conversely, points that fall below the dashed line correspond to regions with a net decrease in presynaptic terminal density. Data are color coded according to age, as indicated in the legend.

In cultured neurons, mobile puncta are observed either budding off of existing synapses to create new synapses or merging with existing synapses to build larger synapses^34^. Division of existing synapses can serve to increase connectivity between two neurons^34,42^, while merging of synapses may enhance synapse strength during development *in vitro*^43^. To determine whether presynaptic terminal division and consolidation contribute to CC synapse development *in vivo*, we examined time-lapse movies of synaptophysin-tdTomato puncta. Some presynaptic terminals divided into multiple puncta (Fig. 4A, Supplementary Fig. S5A and Supplementary Movie 2), and other puncta coalesced to form single puncta (Fig. 4B, Supplementary Fig. S5B and Supplementary Movie 3). When rates of division and consolidation were compared over time, there was no significant age-dependent difference in rates of division and consolidation (Fig. 4C, Supplementary Table 1**;** division: *χ*^2^ (2, 21) = 2.65, p = 0.2657 and consolidation: *χ*^2^ (2, 21) = 2.42, p = 0.2982 via Kruskal-Wallis tests with Dunn-Sidak multiple comparisons corrections). Thoughout development, rates for both process were similar (Fig. 4C, Supplementary Table 1; *F*(1, 21) = 3.99, p = 0.0588, repeated measures ANOVA). However, many individual imaging fields experienced net gains or losses of presynaptic terminals through division and consolidation (Fig. 4D). These observations suggest that division and consolidation contribute to synapse formation and elimination *in vivo*.

## Discussion

Here, we described longitudinal and rapid dynamics of CC presynaptic terminal development *in vivo*. The development of synapses is an important step in the establishment of appropriate neuronal circuits, but relatively little is known about the dynamics of synapse development in the neocortex *in vivo*. This is especially true for long-range projections, such as the corpus callosum. In addition, it is unclear the extent to which synapse development occurs with the same time-course from neuron to neuron. To begin to address these issues, we applied two-photon time-lapse imaging to presynaptic terminals of CC projection neurons during the second and third weeks of postnatal development. To understand the dynamics of synapse formation and elimination, CC presynaptic terminals within the same volumes of cortex were imaged repeatedly over time-scales ranging from minutes to weeks. We found that presynaptic terminal formation and elimination occur over varying time-scales and extents within different axons. In addition, some CC presynaptic terminals were surprisingly mobile, and this mobility decreased with increasing age, possibly corresponding to increasing stabilization of boutons as synaptic connections mature. Finally, presynaptic terminals divided to increase the number of puncta and combined to create fewer puncta, and on average, the rates of these two processes were balanced throughout development. The short-term dynamics that we observed may provide mechanisms for shaping synaptic connectivity as CC axons both form initial synaptic contacts with postsynaptic partners and rearrange or modify connectivity patterns and strengths during development.

What is the role of fusion of multiple presynaptic puncta into fewer boutons and fission of presynaptic puncta into several boutons? Our analysis indicates that, on average, division of existing terminals is balanced by consolidation of other boutons, resulting in no net gain in synapse density. Therefore, these processes might alter the local density of synapses or the overall pattern of synaptic connections, without altering the average density. For example, these processes could selectively change the number of synapses formed with particular postsynaptic partners. In support of this idea, during LTP, perforation of postsynaptic densities has been observed, and this is thought to be followed by splitting of the presynaptic terminals, to amplify synaptic connectivity with potentiated neurons^42,44^. In addition, synapse merging has been proposed to enhance synapse strength during development by forming larger, stronger synapses^43^. It is worth noting that movement of presynaptic terminals could similarly play a role in reshaping connectivity patterns if mobile presynaptic terminals move to another site along the axon in order to change postsynaptic partners. It will be interesting in the future to correlate presynaptic terminal division, consolidation and movement with concomitant alterations in postsynaptic structures and partners.

The approach used here overcomes past technical limitations that precluded analysis of the dynamics of synapse formation by long-range axons in their native environment. Most longitudinal studies of synapses *in vivo* were performed in mature mice, using analysis of filled dendritic spines or axonal swellings^6,45–47^. Axonal swellings are not a reliable indicator of small presynaptic terminals, which are common during early development^30,35,48,49^. Similarly, dendrite fills are inadequate for visualizing synapses with dendrite shafts, which are also prevalent during development, and young spines are found without presynaptic terminals^45^. Furthermore, the identities of presynaptic neurons are unclear when imaging filled dendrites, rendering it impossible to specifically study synapses formed by a specific population of neurons or axons. In addition, the number of labeled CC axons is not identical from animal to animal, making it difficult to examine developmental changes in CC synapse density with cross-sectional analysis of fixed tissue, where the same animal with the same labeled projections cannot be evaluated at different ages. Importantly, our method should be applicable to any long-range projection.

In this study, we specifically labeled presynaptic terminals using a well-established presynaptic marker, synaptophysin. Synaptophysin reliably localizes to presynaptic terminals, even when tagged with fluorescent proteins^50^, such as tdTomato, making it an ideal marker for use in these experiments. Here, synaptophysin puncta colocalized with synapsin and dendrite shafts or spines, lending support to the idea that most of the presynaptic terminals imaged were *bona fide* synapses with postsynaptic partners. However, it is worth noting that, in cultured neurons, a small fraction of presynaptic terminals can be observed without postsynaptic partners^34^. Therefore, we have conservatively referred to synaptophysin-labeled puncta as presynaptic terminals or boutons rather than synapses. Attempts to perform *in vivo* imaging of synaptophysin-tdTomato in double transgenic Thy1-YFP/synaptophysin-tdTomato mice were unsuccessful since YFP expression was not high enough to reliably image connections with labeled cells at young ages when synapse formation occurred at the highest rates; however, future studies could combine our AAV-Cre injection in one hemisphere (to label CC axons) with *in utero* electroporation to express fluorescent postsynaptic markers in the opposite hemisphere.

There are two major strategies for expressing synaptic markers in CC axons: (1) unilateral injection of virus combined with imaging contralateral to the injection, or (2) *in utero* electroporation of cells in one hemisphere combined with imaging of the non-electroporated hemisphere. In our experience, both approaches work for live imaging of CC presynaptic terminal development (our unpublished observations). With virus injection, some pups from a single litter can be kept as baseline controls, with no exposure to surgery or viral injection, to verify that these procedures do not affect overall development, while *in utero* electroporation exposes all pups in a litter to a substantial surgical procedure. In addition, we found that using AAV-Cre in stop-flox synaptophysin-tdTomato mice resulted in more consistent levels of fluorescence and reduced over-expression compared to that achieved with *in utero* electroporation with synaptophysin cDNA (our unpublished observations), likely because the AAV-Cre approach expresses synaptophysin from the genome rather than cDNA. Conversely, the major advantage of *in utero* electroporation is that neurons in a specific cortical layer can be labeled. Since a large majority (around 80%) of CC synapses are derived from layer 2/3 pyramidal neurons^4^, we expect that most of the presynaptic terminals that we imaged were formed by layer 2/3 neurons, as well. *In utero* electroporation with Cre-GFP cDNA is also possible, but we did not evaluate this approach since AAV-Cre requires fewer surgical procedures. Finally, we also evaluated intracranial injection of HSV encoding synaptophysin-YFP; however, this method produced more variable expression levels from neuron to neuron than use of Cre-containing virus with synaptophysin-tdTomato flox mice (data not shown). As a result of the above factors, we chose to apply the Cre-GFP AAV/stop-flox synaptophysin-tdTomato mouse strategy for the experiments presented here.

To analyze CC synapse development, we used time-lapse and longitudinal imaging. Our data underscore the importance of understanding both the short-term (e.g. over minutes) and long-term (e.g. over days to weeks) dynamics of events during synapse formation and elimination. Fixed tissue imaging can be misleading with regard to the rates of synapse formation and elimination and synapse stability. For example, since synapse formation and elimination occur simultaneously, formation and elimination of equal numbers of synapses would appear as no change when fixed tissue is analyzed. Moreover, the high degree of neuron to neuron variability that we observed in our longitudinal studies suggests that, in order to understand these processes, it is ideal to obtain time-course data longitudinally, from the same mouse on successive days. In addition, without time-lapse imaging, it is impossible to understand the extent to which new synapses are formed *de novo* or from splitting of existing synapses.

Another scenario that illustrates the importance of concurrent time-lapse and longitudinal imaging is analysis of synapse turn-over. For longitudinal imaging of spine dynamics, analysis of turnover is frequently presented, reporting percentages of spines that appear, disappear or are stable^51^; however, we did not include rates of presynaptic turn-over in our longitudinal analyses. This analysis was not included since the bouton movement that we saw in our time lapse imaging confounds interpretation of longitudinal turn-over data. For example, at the average velocities and distances traveled by mobile presynaptic puncta (Fig. 3), a given bouton could move on the order of 20-40 μm in an hour. If a presynaptic terminal moved tens of microns between 24 h longitudinal imaging intervals, this one mobile bouton would be counted as addition of one synapse and loss of another, resulting in overestimation of synapse turn-over.

Ultimately, the approach presented here will be useful for determining how sensory experience affects the dynamics of CC synapse development. It is well-established that formation and refinement of CC axon projection patterns depends on sensory input and neuronal activity^6,12,14,15,18,19,22,52,53^. CC projections are initially established exuberantly then refined to their mature patterns^6,15,21^. Axonal arbors appear to reach their mature region- and layer-specific patterns toward the end of the second postnatal week in mice and rats^18,19,23,54^. Interestingly, in rodent somatosensory cortex, maturation of the intrinsic firing properties of CC neurons occurs between P10-17, and this maturation modulates CC axon patterning^55^. Proper CC projection patterning depends on presynaptic tetanus-toxin-sensitive release, presumably of neurotransmitter, and on depolarization of the CC neuron^6,18,54^. Moreover, the balance of cortical activity between the two hemispheres appears to be vital and dependent on neuronal activity since unilateral disruption of intrinsic spiking and strabismus disrupt callosal projection patterning and receptive field properties^12,19^. Analysis of presynaptic puncta at P10 suggests that early synapse density is unaffected by activity or experience^19^; however, it remains unclear how activity and sensory input affects synapse formation and elimination after P10, when the bulk of both formation and elimination are thought to occur.

Our *in vivo* imaging strategy will also be valuable for determining how CC synapse development is disrupted in neurodevelopmental diseases in which CC connectivity is abnormal, including autism, schizophrenia, childhood PTSD, ADHD, dyslexia, epilepsy and Tourette syndrome^1–3^. In such diseases, CC development could be altered by increasing or decreasing synapse formation and/or elimination. In addition, the rate, extent, or duration of these processes could be altered. When combined with mouse models of neurodevelopmental diseases, coordinated longitudinal and rapid time-lapse *in vivo* imaging of synaptophysin-labeled boutons will be a powerful approach for identifying disease-related changes in synapse development.

## Methods

### Mice

All animal procedures were performed according to protocols approved by the Case Western Reserve University Institutional Animal Care and Use Committee. Rosa-CAG-LSL-synaptophysin-tdTomato-WPRE (Ai34D; Stock #012570, Bar Harbor ME, USA)^33^, *Cx3cr1*^*GFP/GFP*^ (stock #5582)^56^ and Thy1-YFP-H (stock #3782)^57^ mice were obtained from the Jackson Laboratory (Bar Harbor, ME). Thy1-YFP-H and Rosa-CAG-LSL-Synaptophysin-tdTomato-WPRE strains were crossed to obtain double heterozygous mice. For all experiments, mice were analyzed from at least 3 separate litters.

### Intracranial virus injection

Replication-deficient AAV1 expressing Cre-GFP under control of the human synapsin promoter was obtained from the University of Pennsylvania Vector Core (University Park, Pennsylvania). At P4, animals were cold-anesthetized, and 1 µl virus was injected intracranially over approximately 60 s, at 2 mm to the left of the sagittal suture and 2 mm posterior to the coronal suture at a depth of 1 mm past the surface of the skull. Incisions were closed with cyanoacrylate surgical glue (Vetbond, 3 M). When respiration and movement returned to normal, pups were returned to their dams.

### Window implantation

Mice were implanted with cranial windows at P10 using a modification of established adult cranial window protocols^58,59^. Briefly, mice were anesthetized with nebulized isoflurane (2% induction, 1.5% maintenance) in 30% O_2_ / 70% air. Body temperature was monitored and maintained between 36.5–38 C, and carprofen and dexamethasone were administered at 5 mg/kg and 0.1 mg/kg, respectively. Hair, skin and periosteum were removed from the skull, and a 2 mm round craniotomy was performed with a #0 dental burr. Craniotomies were positioned to allow imaging of primary somatosensory cortex^60^. A 3 mm round glass coverslip (Warner Instruments) was placed over the craniotomy and attached with cyanoacrylate glue (Vetbond, 3 M). Dental acrylic (Lang Dental) sealed the edges of the coverslip and skin. A small metal stabilization bar was implanted to later secure the mouse to the microscope stage for imaging. After recovery from anesthesia, pups were returned to their mothers or to foster mothers. Use of experienced outbred foster mothers reduced cannibalism. Cranial window implants were well-tolerated, did not appear to interfere with activity or development of the mice, and did not induce inflammation (Supplementary Fig. 3). If bleeding was observed or windows were unclear, animals were not used for imaging.

### Intravital two-photon imaging

Images were collected daily between P13 and P27. In some cases, a day of imaging was missed for practical or logistical reasons, so some longitudinal time courses may not cover the entire span of P13-27. Mice were anesthetized for intravital imaging with nebulized isoflurane (2% induction, 1.5% maintenance) (Baxter) in 30% O_2_ / 70% air (Airgas), with body temperatures maintained at 37 C with a heated environmental chamber. The head was fixed using the implanted stabilization bar to minimize motion artifacts. Callosal synapses within upper layers of cortex were imaged using a Leica SP5 microscope fitted with a DM6000 stage, a 20X water immersion lens (N.A. 1.0; Leica HCX-APO-L), a 16 W pump laser through a Ti/Sapphire crystal to generate an IR laser beam tuned to 880 nm and with an output power of about 3200 mW (Chameleon, Coherent), and a four channel non-descanned external detector using a filter set separating wavelengths of 400–455 nm, 467–499 nm, 500–550 nm, 565–605 nm. Imaging planes (760 × 760 µm) were collected at 1 µm z-intervals. To obtain xyzt data sets, z-stack imaging was repeated at 30 second intervals for up to 30 minutes. Laser power was set as low as possible to maintain imaging quality and avoid phototoxicity. Fiduciary marks and blood vessel morphology were used to image the same volumes on successive days. In some cases, two distinct brain volumes were imaged within the same mouse on each day at different locations within the same window.

### Data analysis

High-resolution 3D and 4D imaging data sets were analyzed using Imaris (BitPlane, Inc). Images were excluded from analysis if there was bone regrowth within the imaging region. As a result, not all imaged regions could be tracked longitudinally for the entire period of P13-27. Tracking analysis was performed on 3D volumes using Imaris manual tracking mode, identifying the center of puncta in an automated fashion based on the maximum intensity. Coordinates were exported and movement was further analyzed via custom written programs in MATLAB. Instantaneous velocity was calculated as the distance an individual punctum moved between consecutive imaging frames, divided by the time between frames. Only instantaneous velocity values above 0.1 µm/sec were recorded as real movements. Distance was calculated as the sum of the distances an individual punctum moved, for all movements with an instantaneous velocity above 0.1 µm/sec. Displacement was calculated as the distance between a punctum’s positions in the first and last frames of imaging. For both distance and displacement measurements, only puncta that moved at least once (with an instantaneous velocity above 0.1um/sec) during the imaging period were utilized for analysis. Also, both distance and displacement values were normalized to the total time imaged, to account for variable imaging period lengths.

For analysis of size and mobility, puncta were identified and measured from the first frame of imaging by using the Imaris spot identification feature. Puncta diameters were estimated using the Imaris surfaces feature to identify the boundaries of three-dimensional puncta based on signal intensity. Estimated diameters were then calculated from the surface areas identified by Imaris, assuming spherical puncta. To identify a threshold for classifying small and large puncta, the diameters of all puncta were analyzed and fitted using an unbiased Gaussian Mixture Model (gmdistribution.fit) in MATLAB (Supplementary Fig. S4A). The data were best fit by a model with two Gaussians, when compared to one or three Gaussians, based on adjusted R^2^ and residuals for the fits of probability density functions from the model, along with Akaike’s information criteria. The data were then sorted in an unbiased manner using MATLAB’s cluster analysis, based on parameters produced by the GMM model (Supplementary Fig. S4B,C). From histograms of the sorted puncta, 2.5 μm was chosen as the division between the two size groups, with small puncta corresponding to presynaptic terminals with apparent diameters of 0.5–2.5 μm, and large terminals corresponding to estimated diameters greater than 2.5 μm. The apparent sizes of the observed puncta were consistent with previous reports of CC synapse size^7^, although a small number of puncta appeared larger than expected. These largest puncta could correspond to particularly bright puncta that appeared larger due to their high intensities or groups of puncta that are closer than the resolution limit of our imaging system or appear overlapping within Z-projections used for presentation purposes. Only puncta with apparent diameters of 0.5–5 μm were used for further analysis.

Presynapse density changes were determined with the following formula: % change = 100*(bin2-bin1)/bin1. Bins correspond to the average density within the relevant time window for each imaging field (e.g. for the top panel of Fig. 2C, bin 1 = P13-15, and bin 2 = P16-18). For each cortical volume analyzed, the size and position of the imaged field was consistent across all timepoints. The mean intensities of presynaptic terminals in all imaged regions were measured in each image set to verify that changes in density were not due to changes in tdTomato expression or laser power. Images were excluded from analysis if mean intensities were different. For presentation, maximum-intensity Z-projections were made and images were cropped in Imaris, ImageJ or Photoshop. For a given imaging field, volumes used for Z-projections were kept consistent across ages.

Analysis of which age groups experienced significant changes in density was with a Wilcoxon signed rank test. Statistical analysis of the time-course of daily changes in density from P13-17 was via a Kruskal-Wallis test with Tukey-Kramer multiple comparisons correction. Comparison of the percentages of large and small puncta that were mobile was performed with repeated measures ANOVA. Velocities, distances and displacements were analyzed using a Kruskal-Wallis test with a Dunn-Sidak multiple comparison adjustment. Merging was compared to splitting using repeated measures ANOVA. For analysis of age-dependent changes in division or consolidation, Kruskal-Wallis tests were used with Dunn-Sidak multiple comparisons corrections. All error bars are s.e.m.

### Immunohistochemistry

When all live imaging was completed, mice (ages P26-29) were deeply anesthetized via isoflurane inhalation and then transcardially perfused with saline followed by 4% paraformaldehyde. Brains were removed and fixed in 4% paraformaldehyde for 1 hour before being transferred to 30% sucrose for cryoprotection. Frozen brains were then sectioned via Leica CM1850 cryostat at thicknesses of either 10–15 µm or 40 µm. Immunohistochemistry was performed on thin (10–15 µm) coronal sections utilizing rabbit anti-synapsin antibodies (Synaptic Systems, Goettingen, Germany) with goat anti-rabbit secondary antibodies conjugated to Alexa Fluor 488. NISSL staining was performed on 40 µm coronal sections with NeuroTrace (catalog # N-21483, Molecular Probes), according to manufacturer instructions. Sections were mounted with fluoromount containing DABCO (Sigma, St. Louis, MO, USA) and imaged via a C1 Plus confocal system on a Nikon Eclipse Ti-E microscope utilizing 20x Nikon Plan Apo 0.75NA or 40X Nikon Plan Apo 0.95NA objectives.

## Supplementary information

Supplementary movie 1

Supplementary movie 2

Supplementary movie 3

supplementary information

## Data Availability

Data and MATLAB scripts that contributed to this study are available from the corresponding author on request via email.
