# Supplementary material for: Spatio-temporal dynamics of neocortical presynaptic terminal development using multi-photon imaging of the corpus callosum in vivo
Source: Sci Rep. 2019 Oct 1;9:14028. doi: 10.1038/s41598-019-50431-6 (PMC6773694; doi:10.1038/s41598-019-50431-6)
Supplement: Supplementary file 4 — supplementary information [file 41598_2019_50431_MOESM4_ESM.pdf]

Spatio-temporal dynamics of neocortical presynaptic terminal development using multi-photon imaging of the corpus callosum *in vivo*

Teresa A. Evans[1,4], Luke A. Bury[1], Alex Y. Huang[2], Shasta L. Sabo[1,3*]

[1]Departments of Pharmacology and Neuroscience, Case Western Reserve University School of Medicine

[2]Department of Pediatrics, Case Western Reserve University School of Medicine

[3]Department of Biology, Central Michigan University

[4]Current Address: Department of Pediatrics, Stanford University

*Corresponding author, email: sabo1s@cmich.edu

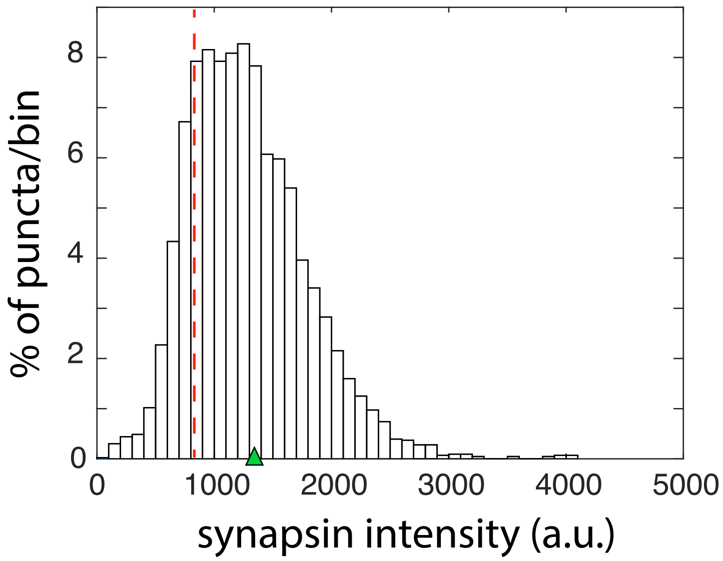

Supplementary Figure S1: Synaptophysin puncta colocalize with synapsin in the contralateral cortex. Histogram of synapsin intensities at synaptophysin-tdTomato presynaptic puncta. Coronal slices were made from the brains of mice that had been imaged in vivo. Slices were immunolabeled with anti-synapsin antibodies, and individual optical sections were imaged and analyzed. Synaptophysin-tdTomato puncta were identified using an automated ImageJ macro, then intensities of synapsin immunolabeling within puncta were measured and compared to neighboring background fluorescence. Data represent the percentage of puncta at each synapsin intensity level. Red dashed line, background fluorescence intensity within the same images (859.4 +/- 51.7). Green arrow, mean synapsin intensity at synaptophysin-tdTomato puncta (1350.0 +/- 72.5). Intensities at synaptophysin-tdTomato were significantly higher than background (paired t-test: $p = 1.187 \times 10^{-8}$, $t(19) = 9.5$; n = 20 images).

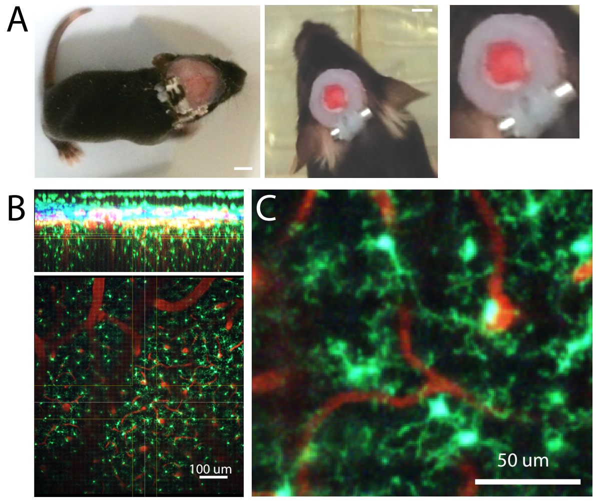

Supplementary Figure S2: Window implantation and in vivo imaging in young mice are well-tolerated and do not cause inflammation, as shown by microglial morphology. (A) Juvenile mice at two ages with implanted glass windows and stabilization bars for in vivo imaging. Left, beginning of imaging (P14). Middle, end of imaging (P27). Right, enlargement of window region from the older mouse. Scale bars, approximately 2mm. (B) CX3CR1 heterozygous mouse showing normal ramified microglial architecture (green) in a P14 mouse at 4 days after window implantation. Images were collected in living mice, through implanted windows. Blood vessels were labeled by IV injection of rhodamine conjugated dextran (red). Top, X-Z projection. Bottom, X-Y projection. (C) Higher magnification of microglia showing details of ramified structure, indicating lack of parenchymal inflammation.

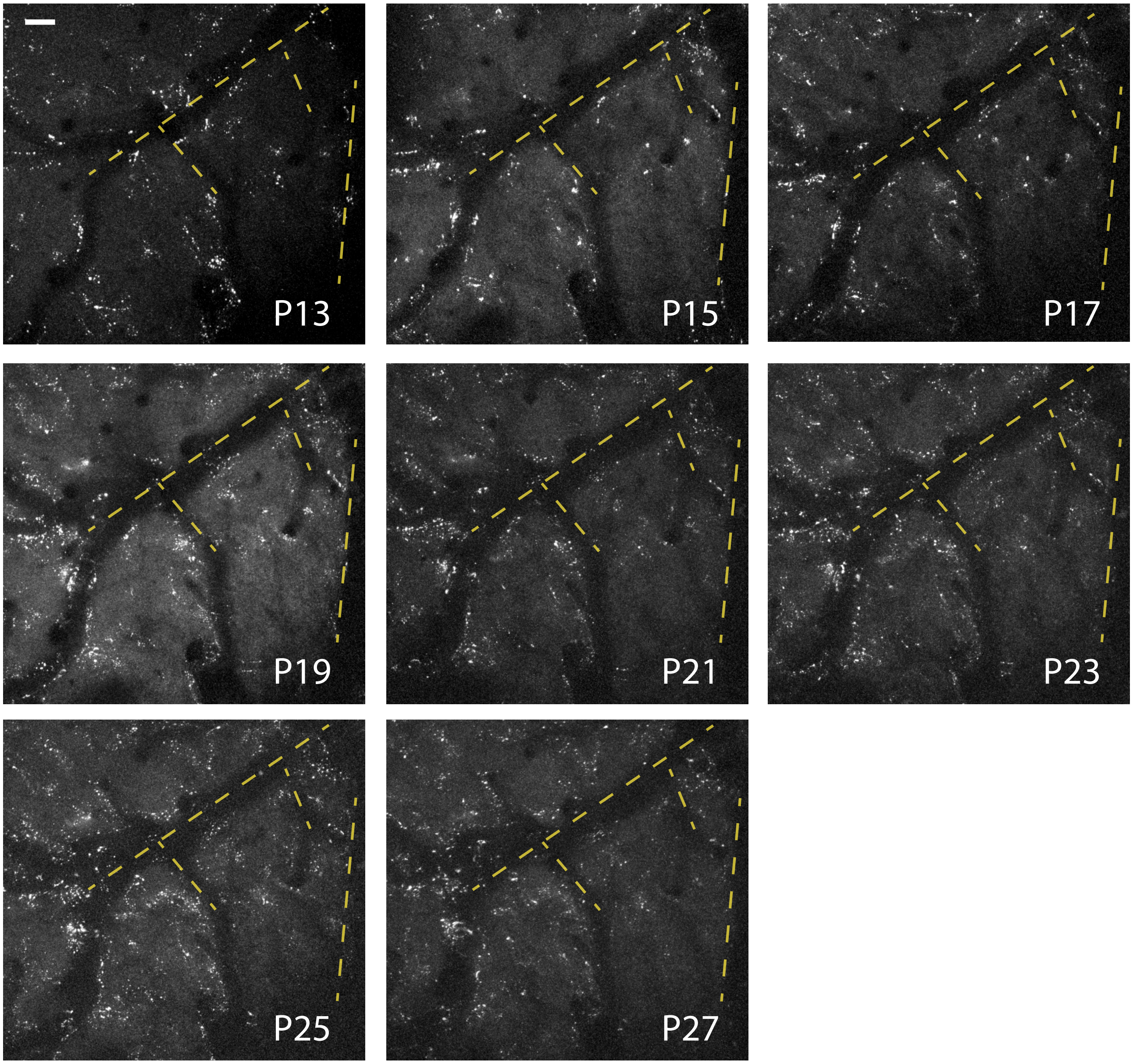

Supplementary Figure S3: Daily longitudinal imaging of the same brain volume. The absence of background fluorescence in blood vessels, combined with distance from the cortical surface, allowed recognition of the same brain volumes for imaging on consecutive days. Z-stacks of the same volume were collected and analyzed on each day. The images shown are maximum intensity Z-projections of the stacks. The same brain volume was imaged and analyzed from P13-27, and every other day of imaging is shown for 1 mouse. Dashed lines indicate the same blood vessels at each timepoint. Scale bar, 50µm.

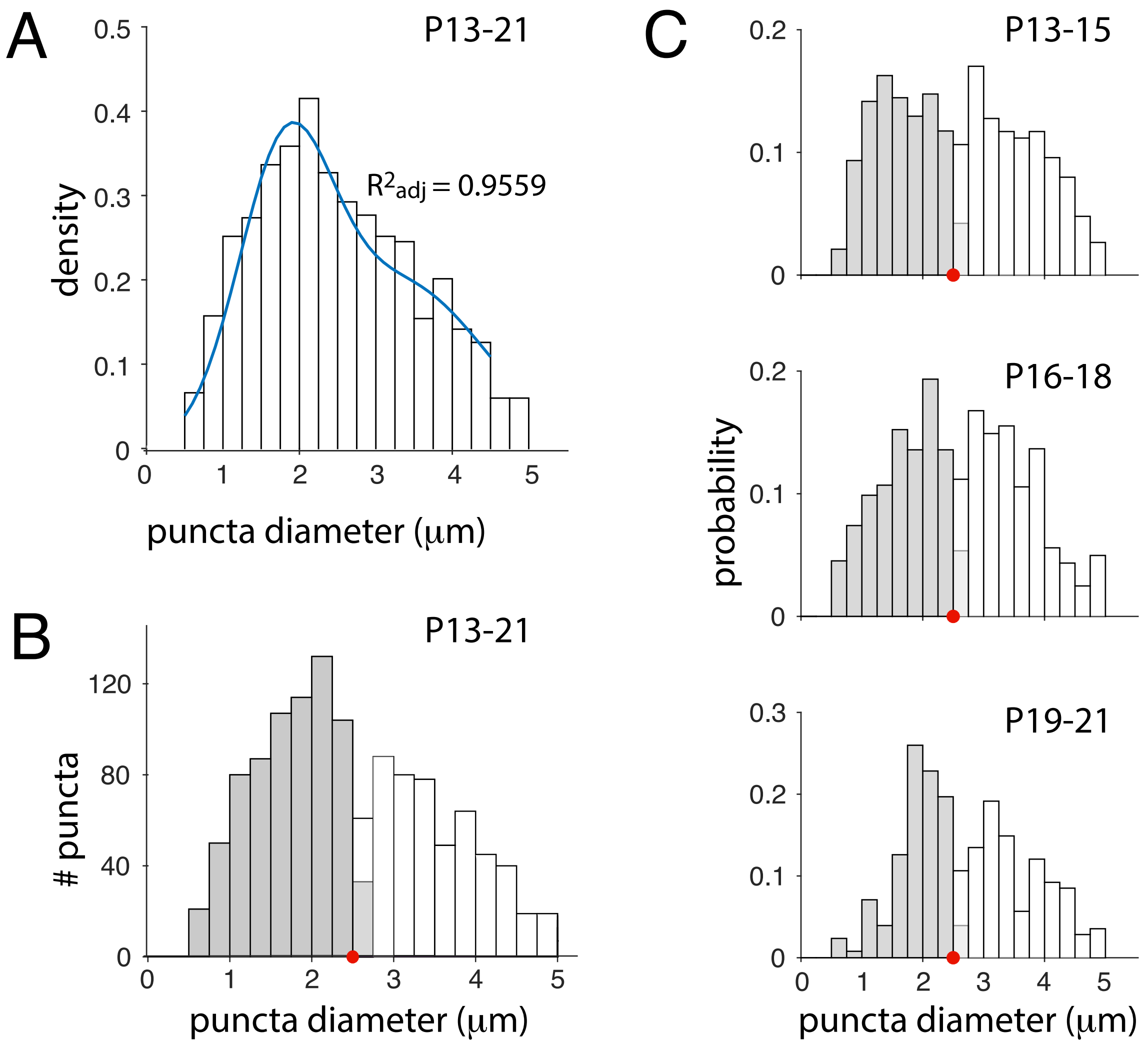

Supplementary Figure S4: Distribution of estimated presynaptic puncta diameters. (A) Probability density histogram of the apparent diameters of all puncta between 0.5-5μm for all age groups analyzed (P13-P21). The data were fitted with two Gaussians (blue) using an unbiased Gaussian mixture model (GMM). Fitting with two Gaussians maximized R^2 (0.9655) and adjusted R^2 (0.9559) and minimized SSE and RMSE, when compared to fits with one or three Gaussians (adjusted R^2: 0.8754 and 0.9454 for one and three curves, respectively). (B) Histograms of puncta sizes for all ages combined, clustered by size. Small (grey) and large (white) size histograms were generated by subjecting the data to cluster analysis, based on the optimized GMM parameters from A. Based on the analyses in A and B, a diameter of 2.5μm (red dot) was chosen as the cut-off for segregation into large and small puncta. (C) Probability histograms of puncta sizes for three age groups: P13-15, P16-18 and P19-21. Data were clustered into small (grey) and large (white) groups based on the GMM parameters derived in A. Segregation of small and large puncta based on a 2.5μm diameter cut-off was reasonable for all ages analyzed. In addition, adjusted R^2 was high (P13-15: 0.9262; P16-18: 0.8719; P19-21: 0.8681) for fits of all age groups with two Gaussians.

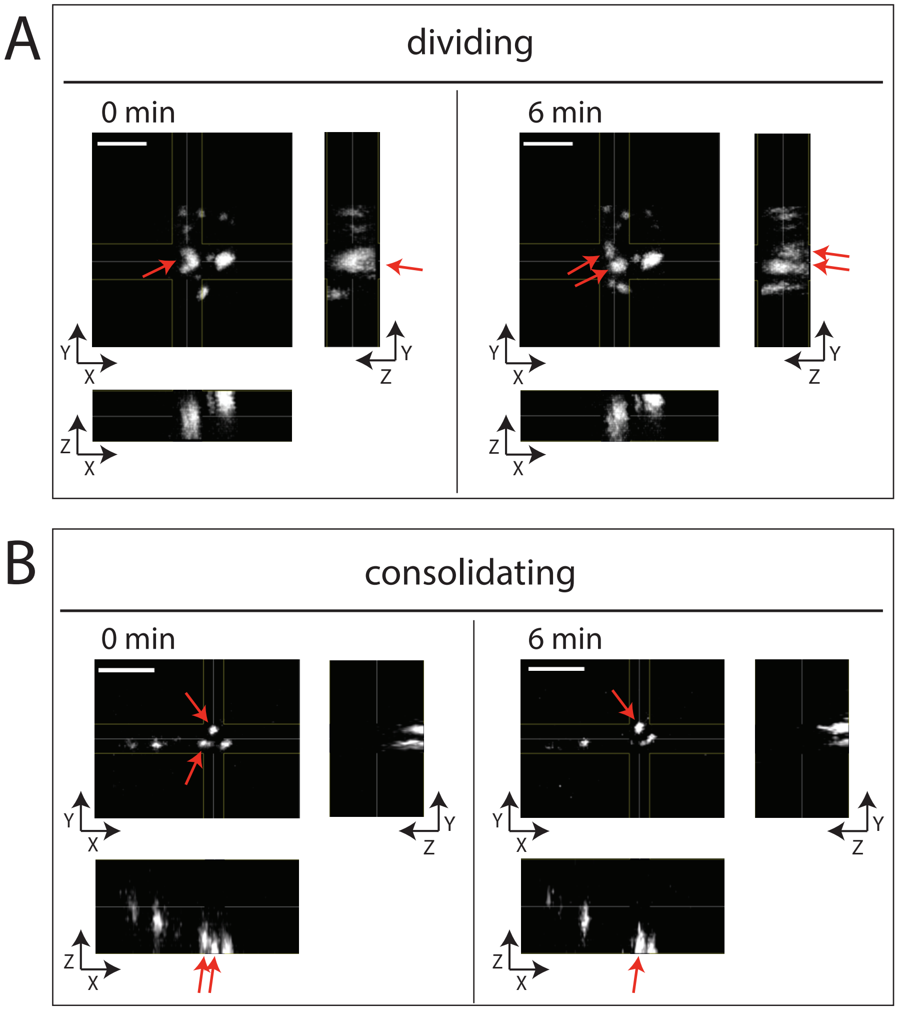

Supplementary Figure S5. Three-dimensional representations of dividing and consolidating presynaptic puncta. X/Y, X/Z and Y/Z planes of the time lapse image sequences presented in Fig. 4A-B were derived from maximum intensity projections for the indicated times. X/Z and Y/Z projections were made for the areas marked by cross-hatches in the X/Y projections. (A) The Y/Z planes verify division of puncta (red arrows) observed in X/Y planes and in the time lapse sequence in Fig. 4A. (B) Similarly, X/Z planes verify the consolidation of puncta (red arrows) observed in the X/Y planes and the time lapse sequences in Fig. 4B.

Supplementary Table 1: Values for data presented in Figures 3 and 4, represented as mean +/- s.e.m.

	P13-15 (N = 3 mice)	P16-18 (N = 4 mice)	P19-21 (N = 4 mice)
distance moved (µm) in one minute	0.675 +/- 0.122<br>N = 16 puncta	0.763 +/- 0.076<br>N = 42 puncta	0.313 +/- 0.040<br>N=29 puncta
net displacement (µm) in one minute	0.583 +/- 0.113<br>N = 16 puncta	0.633 +/- 0.091<br>N = 42 puncta	0.273 +/- 0.042<br>N = 29 puncta
instantaneous velocity (µm/s)	0.141 +/- 0.001<br>N = 35 movements	0.128 +/- 0.004<br>N = 64 movements	0.126 +/- 0.004<br>N = 47 movements
% divided in one minute	0.0870 +/- 0.0323<br>N = 5 movies	0.1490 +/- 0.0398<br>N = 10 movies	0.0606 +/- 0.0157<br>N = 9 movies
% consolidated in one minute	0.1803 +/- 0.0590<br>N = 5 movies	0.2544 +/- 0.0375<br>N = 10 movies	0.1532 +/- 0.0399<br>N = 9 movies

Supplementary Movie 1: Example of movement and tracking of synaptophysin-labeled puncta.

Time-lapse movie of synaptophysin-tdTomato (*yellow*) in a P14 mouse. Tracks of puncta movement are shown over a period of 7.5 minutes. Tracks are color-coded for time. Movie is 150x real time, with one frame obtained every 30 seconds.

Supplementary Movie 2: Example of division of synaptophysin puncta.

Time-lapse movie of synaptophysin-tdTomato (*white*) showing an example of one large punctum dividing into two puncta of unequal sizes in a P18 mouse. Total duration of imaging is 10 minutes. Movie is 150x real time, with one frame obtained every 30 seconds.

Supplementary Movie 3: Example of consolidation of synaptophysin puncta.

Time-lapse movie of synaptophysin-tdTomato (*white*) showing an example of two small puncta consolidating into a single larger punctum that is stationary in a P18 mouse. Total duration of imaging is 10 minutes. Movie is 150x real time, with one frame obtained every 30 seconds.
